# Smartphone-Derived Seismocardiography: Robust Approach for Accurate Cardiac Energy Assessment in Patients with Various Cardiovascular Conditions

**DOI:** 10.3390/s24072139

**Published:** 2024-03-27

**Authors:** Amin Hossein, Elza Abdessater, Paniz Balali, Elliot Cosneau, Damien Gorlier, Jérémy Rabineau, Alexandre Almorad, Vitalie Faoro, Philippe van de Borne

**Affiliations:** 1 Laboratory of Physics and Physiology, Université Libre de Bruxelles, 1050 Brussels, Belgium; 2 Cardio-Pulmonary Exercise Laboratory, Faculty of Motor Sciences, Université Libre de Bruxelles, Erasme Campus, Anderlecht, 1070 Brussels, Belgium; 3 Department of Cardiology, Erasme Hospital, Université Libre de Bruxelles, 1050 Bruxelles, Belgium; 4 Heartkinetics S.A., 6041 Charleroi, Belgium; 5 Heart Rhythm Management Centre, Postgraduate Program in Cardiac Electrophysiology and Pacing, Vrije Universiteit Brussel, Universitair Ziekenhuis Brussel, 1050 Brussels, Belgium

**Keywords:** smartphone, e-health, cardiac kinetic energy, telemedicine, ballistocardiography, cardiovascular screening

## Abstract

Seismocardiography (SCG), a method for measuring heart-induced chest vibrations, is gaining attention as a non-invasive, accessible, and cost-effective approach for cardiac pathologies, diagnosis, and monitoring. This study explores the integration of SCG acquired through smartphone technology by assessing the accuracy of metrics derived from smartphone recordings and their consistency when performed by patients. Therefore, we assessed smartphone-derived SCG’s reliability in computing median kinetic energy parameters per record in 220 patients with various cardiovascular conditions. The study involved three key procedures: (1) simultaneous measurements of a validated hardware device and a commercial smartphone; (2) consecutive smartphone recordings performed by both clinicians and patients; (3) patients’ self-conducted home recordings over three months. Our findings indicate a moderate-to-high reliability of smartphone-acquired SCG metrics compared to those obtained from a validated device, with intraclass correlation (ICC) > 0.77. The reliability of patient-acquired SCG metrics was high (ICC > 0.83). Within the cohort, 138 patients had smartphones that met the compatibility criteria for the study, with an observed at-home compliance rate of 41.4%. This research validates the potential of smartphone-derived SCG acquisition in providing repeatable SCG metrics in telemedicine, thus laying a foundation for future studies to enhance the precision of at-home cardiac data acquisition.

## 1. Introduction

Telemedicine is revolutionizing healthcare by enabling remote care, education, and monitoring through digital communication technologies. It is beginning to play a central role in transitioning from predominantly curative to preventive medicine. It allows patient autonomy and involvement in healthcare plans. This approach benefits elderly patients and those with chronic conditions like heart failure, offering a cost-effective and patient-centered care mode [1]. Despite its many advantages, telemedicine faces challenges such as technological barriers, privacy concerns, and the need for specialized training for healthcare professionals and patients. These challenges can impact the effectiveness and reach of telemedicine services, particularly in remote and underserved areas [2]. Telemedicine has become increasingly important in cardiac care because of the global burden of cardiovascular disease. It provides a platform for remote monitoring and managing cardiac patients, essential for timely intervention and continuous care. This approach is particularly beneficial for both primary and secondary prevention strategies. Primary prevention focuses on preventing the onset of heart diseases in high-risk individuals through lifestyle modification and risk factor management, which can be effectively monitored and encouraged remotely. Secondary prevention targets patients who have experienced cardiac events, providing them with continuous monitoring and immediate medical response to prevent recurrence. However, cardiac telemedicine also encounters specific challenges, such as ensuring the accuracy of remote monitoring technologies and the reliability of data transmission [3].

Seismocardiography (SCG) emerges as a promising technique in this context. SCG, which records heart-induced chest vibrations, offers a non-invasive and potentially cost-effective method for cardiac monitoring [4,5]. Integrating SCG into telemedicine could address many challenges in cardiac care, particularly in remote and continuous monitoring. The advent of smartphones has further expanded the potential of SCG in cardiac telemedicine. Smartphones equipped with SCG capabilities could democratize access to advanced cardiac monitoring tools, making it easier for patients to engage in their health management. Integrating smartphone technology with SCG could significantly enhance the reach and effectiveness of cardiac telemedicine, thus offering a promising solution to its current challenges. 

Recent studies have explored this potential with mixed outcomes. For instance, Ramos-Castro et al. demonstrated the viability of smartphones in recording cardiac activity, highlighting similarities with ECG signals despite certain limitations [6]. The study of beat-by-beat heart rate (HR) estimation, utilizing smartphone-based SCG primarily through acquiring angular rates via built-in gyroscopes, yielded positive results [7,8,9]. Furthermore, the potential of smartphone-derived SCG in identifying cardiac arrest scenarios was investigated, underscoring its future possibilities [10]. Mehrang et al.’s investigation into the use of smartphone-derived SCG signals for diagnosing myocardial infarction, which achieved accuracies of 74% and 78% with random forest and SVM classifiers, respectively, underscores the burgeoning potential of smartphone technology in cardiac analysis [11]. This potential is further evidenced by the MODE-AF study, which leveraged a smartphone-based algorithm to distinguish atrial fibrillation (AF) from sinus rhythm with remarkable sensitivity (95.3%) and specificity (96.0%) [12]. Moreover, the same technique was used to detect acute decompensated heart failure (ADHF) with moderate accuracy in a subsequent study involving 300 cardiac patients, highlighting the versatility of smartphone technology in cardiac care [13]. Conversely, challenges in standardizing smartphone-based SCG for clinical applications were outlined, stressing the gap between research and bedside application [14].

Building upon this foundation, another dimension of smartphone-based cardiac monitoring was explored through the self-application of SCG technology. A study involving 182 elderly patients revealed that self-applied measurements could achieve over 96% sensitivity and 92.9% specificity, suggesting that patients can reliably monitor their cardiac health with minimal professional intervention [15]. However, it is important to note that, while the study validated the algorithm’s classification reliability, it did not delve into the direct comparison of measurement reliability between physician-applied and self-applied methods.

The positive outputs of these studies on the application of smartphone-derived SCG in managing cardiovascular diseases highlight an innovative shift toward more accessible and user-friendly cardiac monitoring techniques. Despite the promising results, there remains a gap in the literature regarding a formal, comprehensive comparison between SCG from validated devices and those obtained via smartphones, particularly in patients with confirmed cardiac pathologies. Our study aims to bridge this gap by not only comparing these two modalities but also examining the differences between clinician-applied and patient-self-applied SCG. This comparison is crucial to validate the efficacy and reliability of smartphone-derived SCG in clinical settings.

**Figure 1:**
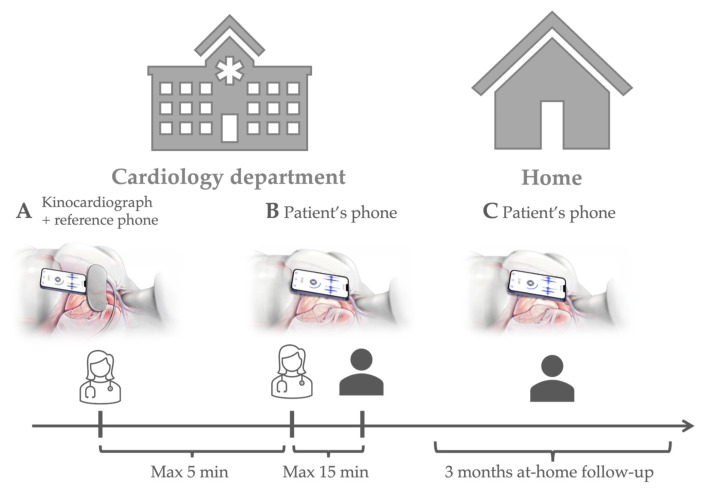
General overview of the study. The patients are instrumented with the kinocardiograph [16] and a reference Apple smartphone (iPhone SE 2020 with an application acquiring accelerations and angular rates at a sampling rate of 100 Hz). A mobile application was also installed on the patient’s personal smartphone. The smartphone was placed on the manubrium of the sternum, below the clavicle of the patient, who was asked to rest in the supine position for 5 min and to breathe normally before the start of the acquisitions. Then, the investigator performed an acquisition with the kinocardiograph and the reference smartphone synchronously (**A**), followed by a similar recording with the patient’s personal smartphone; thereafter, this recording was repeated less than 15 min later by the patient themselves (**B**). The patient was then instructed to perform measurements at home with their own smartphone three times a week for three months (**C**).

Therefore, this study evaluated the reliability of kinetic energy metrics derived from the SCG acquired using a smartphone. Additionally, this study also evaluated the compliance of patients with do-at-home SCG measurements with a smartphone. To do so, a new method to compute kinetic energy based only on an SCG signal is presented. We focused on measuring resting-state activity in patients with diverse cardiovascular diseases at three distinct times to determine the reliability of these metrics under different conditions. This included the following: (A) Simultaneous measurements, using a validated device compared to a commercial smartphone, conducted by a trained healthcare professional; (B) Intrasession reliability using another commercial smartphone first by a healthcare professional and then by the patient; (C) Patients conducting at-home measurements using their smartphones (Figure 1).

## 2. Materials and Methods

### 2.1. Protocol and Participants

A total of 220 subjects have been recruited in the ambulatory routine of the cardiology department at the Erasme Hospital (Brussels, Belgium). The study protocol complied with the Declaration of Helsinki, was approved by the local Ethics Committee (Hôpital Erasme—CCB: B4062020000186), and was registered on ClinicalTrials.gov (NCT04772807). The Belgian Federal Agency authorized the prototype of the hardware device, namely the kinocardiograph, used in this clinical trial for Medicine and Health Products (FAMHP-80M0861). Written informed consent was obtained from each participant before the experimental testing procedure. All patients underwent a medical history assessment and a physical examination before their inclusion in the study.

The protocol consisted of the following: (A) A simultaneous measurement with the kinocardiograph and with an application running on a standard Apple smartphone (iPhone SE 2020), performed by the healthcare provider at the hospital; (B) A measurement performed also by the healthcare provider just a few minutes later, using the patient’s personal smartphone, and repeated less than 15 min later by the patient themselves; (C) Measurements performed by the patient with their own smartphone at home three times a week, for three months. In the cardiology department (Phases A and B), measurements were taken with patients lying in a hospital bed, their heads on a pillow. At home (Phase C), patients were instructed to conduct the measurement first thing in the morning while still in bed.

**Figure 2:**
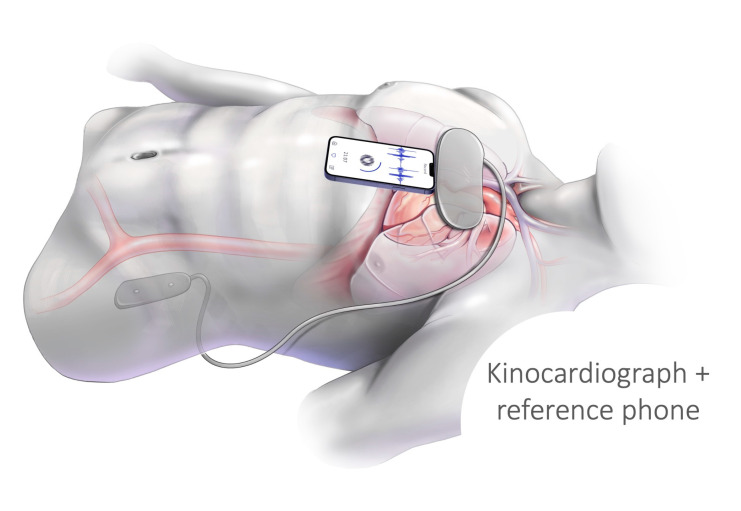
Schematic representation of simultaneous acquisition using a kinocardiograph and a commercial smartphone.

The setup for phase (A) is illustrated in Figure 2. The measurements of the Kinocardiograph and the smartphone were started simultaneously. However, they were not synchronized on a beat-by-beat basis. To participate in phases B and C of the study, the patient’s smartphone had to comply with the following criteria: (1) Have access to the app stores to download the app; (2) Contain accelerometers and gyroscopes. Both Apple and Android systems were included. During at-home periods, if the patient forgot to make a recording, a dedicated nurse would initiate a phone call once a week to provide a reminder. This protocol is illustrated in Figure 1. For each record, the patient was asked to breathe normally and remain still for 3 min in the supine position.

### 2.2. Kinetic Energy Data Analysis

The kinocardiograph is a wearable device with two detectors, one of which is placed over the lumbar region close to the subject’s center of mass and the other on the chest. Each detector contains a microelectromechanical systems (MEMS) accelerometer and gyroscope sensor (LSM6DSL, STMicroelectronics, Geneva, Switzerland) and is attached to the body with standard sticky gel electrodes. The acceleration and angular rates of the sensor were set to ±2 g and ±250 dps, respectively, with a resolution of 0.061 mg/LSB and 4.375 mdps/LSB and an RMS noise of 80 μgHz and 4 mdpsHz with an output bandwidth of 416 Hz. The kinocardiograph is controlled with a smartphone or a tablet connected via Bluetooth and collects a two-lead electrocardiogram (ECG) at 200 Hz (ADS1292R, AD Instruments, Sydney, Australia) together with 3-DOF linear accelerations and 3-DOF rotational angular velocities from the chest (SCG) and the lumbar region. The analysis of angular velocities measured using a chest-mounted sensor is also referred to as gyrocardiography [17,18]. Linear acceleration and angular velocity signals were recorded at 100 Hz. A 4th-order Butterworth IIR filter with 0.5–60 Hz pass band was applied to the ECG signals. The device is described in previous publications [16]. The standard nomenclature [19] was used for SCG signals; the z-axis points are dorsoventral, and the x-axis is mediolateral. To acquire smartphone-derived SCG, a non-commercially available custom application acquiring accelerations and angular rates at a sampling rate of 100 Hz was installed on the smartphones. To ensure this, a timestamp was associated with each sample, and the length of the total samples per record was checked. When the internet is available, the application directly sends the data to a cloud server. Raw SCG signals were filtered using a window-based finite impulse response (FIR) bandpass filter using a Hamming window with cutoff frequencies of 3–50 Hz. The Fred–Harris rule was used to compute the number of taps with a target attenuation of 60 dB. The high-pass cutoff frequency was selected empirically, based on our experience and with the filters used by other groups. The low-pass cutoff frequency was selected at the limit of the Nyquist–Shannon sampling theorem. The kinetic energy (KE) signals were then computed using Newtonian equations. To achieve this, the weights of the sensors were used.

(1) $${KE}_{Lin}=\frac{1}{2}m{(v}_{x}^{2}+v_{y}^{2}+v_{z}^{2})$$

(2) $${KE}_{Rot}=\frac{1}{2}(I_{xx}\omega_{x}^{2}+I_{yy}\omega_{y}^{2}+I_{zz}\omega_{z}^{2})$$

where m is the mass of the sensor; ${KE}_{Lin}$ is the linear kinetic energy; $v_{x}$,$\;v_{y}$, and $v_{z}$ are the orthogonal components of the velocity vector $\vec{v}$; ${KE}_{Rot}$ is the rotational kinetic energy; $I_{xx}$,$\;I_{yy},\;and\;I_{zz}$ are the orthogonal components of the moment of inertia; and $\omega_{x}$,$\;\omega_{y},$ and $\omega_{z}$ are the orthogonal components of the angular velocity $\vec{\omega }$ measured from the gyroscopes. 

Movement artifacts were detected on linear and rotational *KE* signals and corresponded to the time intervals where the signal was greater than a threshold *τ*.

(3) τ=median(KE)+κ ∗ IQR(KE)

where *IQR* is the interquartile range and κ is set to 100. To perform a quality check, the detected windows were then expanded by 1000 ms to ensure that the remaining signal was free of movement artifacts. Only the resulting “clean” signal was considered in the remaining analyses. Then, a low-frequency signal was derived from linear and rotational *KE* to detect the heartbeats’ first set of probable locations. To achieve this, these signals were first filtered by an FIR bandpass filter in a similar way than described previously, with cutoff frequencies of 0.65 and 3.5 Hz. The cutoff frequencies were chosen to reflect the heart rate frequency band. They were then standardized by subtracting the median from the signal and dividing it by the *IQR*. A low-frequency profile signal $S_{lf}$ was then computed by combining the linear and rotational low-frequency signals $S_{lf}^{lin}$ and $S_{lf}^{rot}$:

(4) $$S_{lf}=\frac{\lambda_{lin}*{\;S}_{lf}^{lin}+\lambda_{rot}\;*\;S_{lf}^{rot}}{\lambda_{lin}+\lambda_{rot}}$$

where $\lambda_{lin}$ and $\lambda_{rot}$ were the weights of each signal in $S_{lf}$. In this work, $\lambda_{lin}$ and $\lambda_{rot}$ were both set to 1. Peaks and troughs were detected on the $S_{lf}$ signal and were used as points of interest for detecting the ventricular systole of each heartbeat. A score σ was computed for each peak and trough based on the RMS envelope ($E_{lf}$) of the $S_{lf}$ signal with a window of 600 ms. The initial value was the ratio between the signal $S_{lf}$ and the envelope $E_{lf}$ at the time of the peaks and troughs. Then, the value of a trough was associated with a peak if its position was within an interval between −350 and −100 ms before this peak. These values were multiplied by the peak prominences to obtain the peak scores. The peaks were finally filtered by sorting the scores in descending order and removing the peaks located closer than a distance of 450 ms from a higher score peak. These settings were the same for all patients. 

Windows around the filtered peaks based on an interval between −200 and 300 ms were computed, and the Matrix Profile algorithm was used to find motifs [20,21], i.e., repetitive patterns, in those windows, with a length of 400 ms. These motifs represent the heartbeats. The intervals between these were used to compute the HR. The average HR was the mean of each individual interval between motifs within a recording. The middle of the identified motifs represents the reference points to segment the phases of the cardiac cycle. These settings were the same for all patients. 

Based on these acquisitions, the time integrals of the linear KE (SCG i$K_{Lin}$) were computed in different phases of the cardiac cycle:

- iK_Sys_, which represented the cardiac activity during the systolic phase, and was computed between −100 ms and 100 ms around the reference point.
- iK_Late dia_, which represented the cardiac activity during the late diastolic phase, and was computed between −300 ms and −100 ms before the reference point.
- iK_Early dia_, which represented the cardiac activity during the early diastolic phase, and was computed between 100 ms and 300 ms after the reference point.

**Figure 3:**
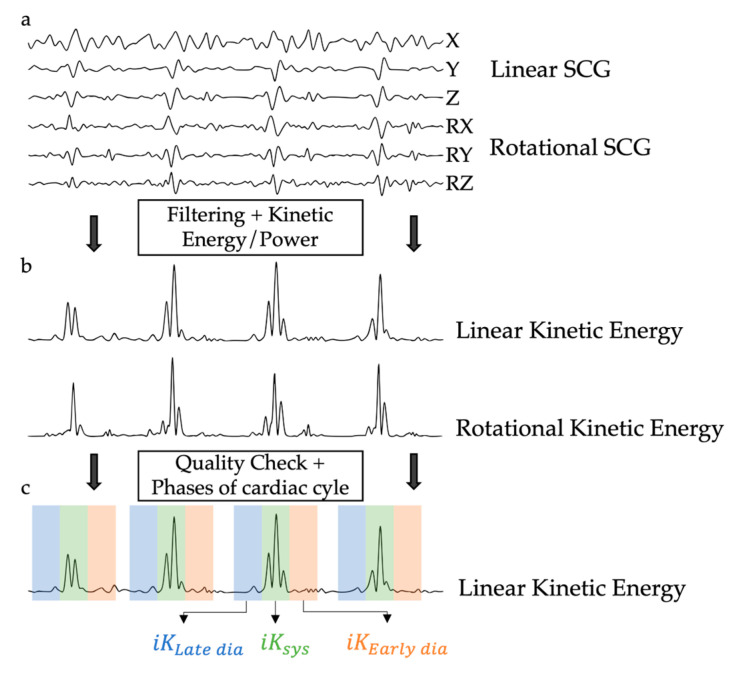
Schematic representation of the kinetic energy computation based on seismocardiography. (**a**) The 6 channels of the SCG signal, 3 linear axes, and 3 rotational axes. (**b**) The Kinetic Energy signal computed from the 6 channels of SCG. (**c**) The Kinetic Energy signal and the segmented phases of the cardiac cycle.

This procedure is summarized in Figure 3. These metrics aim to reflect metrics previously computed based on an ECG segmentation [22]. For each record, the median iK_Sys_, iK_Late dia_, and iK_Early dia_ of all the valid heartbeats within a record were computed and used to compare the records. In previous publications [22,23], rotational kinetic energy was reported and analyzed. However, since it did not yield results that differed from those of linear kinetic energy, this parameter is not independently investigated in the current work. All data analyses were performed offline using a proprietary software toolbox under Python (v3.11.5).

### 2.3. Statistics

#### 2.3.1. Repeatability Analysis

Data are presented as median [Q1; Q3]. The study used a dual-method approach for evaluating metrics obtained with a smartphone compared to those acquired with the kinocardiograph, as well as for assessing the differences between recordings made by a trained clinician versus a patient. 

Firstly, Bland–Altman (BA) plots were generated for each metric of interest [24]. The measures were considered similar when the BA plots displayed (1) an absence of a positive or negative trend and (2) limits of agreements (LoA) within intervals that define clinical goals to determine if the differences are too wide or sufficiently narrow for the specific purpose-tested [24]. The BA LoA, defined as ±1.96 standard deviations (STD) between both measurements with a 95% Confidence Interval, should allow more than 95% of the differences to lie within the LoA in case of normal distribution. To ensure this, a Liliefors test was performed first to assess if the differences followed a normal distribution. In addition, we verified if at least more than 90% of the differences lied within the LoA.

The agreement intervals were determined based on the variation observed between patients with heart failure vs. a matched control group, as reported in a previous study [22]. Consequently, the agreement intervals for each metric are a 67% variation in iK_Sys_, 35% in iK_Late dia_, and 7% in iK_Early dia_ in terms of the percentage change. The reference values are reported in Appendix A. Therefore, measurements were considered comparable when the limits of agreement, defined as ±1.96 standard deviations between both measurements with a 95% Confidence Interval, aligned within these pre-established clinical agreement intervals. We investigated the presence of fixed biases between each metric by applying a 1-sample *t*-test in case of a normal distribution or a 1-sample Wilcoxon signed rank test otherwise.

Secondly, we used intraclass correlation coefficients (*ICC*) to assess the reliability of kinetic energy metrics. Multiple variants of ICC exist, each with different advantages and limitations, first presented by Shrout et al. [25] and further developed by McGraw et al. [26]. The specific form used here is a two-way mixed model without interaction (Equation (5)). This model measures the absolute agreement of measurements made under the fixed level of the measurement factor.

(5) $${ICC}_{c}=\frac{{MS}_{p}-{MS}_{e}}{{MS}_{p}+\left(k-1\right){MS}_{e}+\frac{k}{n}({MS}_{c}-{MS}_{e})}$$

where *MS_p_* is the between-sessions ([kinocardiograph vs. phone measure] or [HCP recording vs. patient recording]) mean square representing the variability between sessions, *MS_e_* is a residual mean square traditionally referred to as mean square error of measurements, ${MS}_{c}$ is the between-participants mean square representing the variability between participants, n is the number of participants, and k is the number of measurements. A high value of ICC shows a fair degree of agreement between the devices [26]. An ICC above 0.7 is considered satisfactory; above 0.8 is excellent.

#### 2.3.2. At-Home Measurements Patient Compliance

We assessed the discrepancy between the actual number of at-home recordings and the expected number. This was quantified as a percentage of the recordings actually completed. The data were categorized based on patient age groups: under 45, between 45 and 75, and over 75 years. To determine if there were statistically significant differences between these groups, an ANOVA test was conducted. In instances where the ANOVA results were significant, compliance among different age groups was compared using either an independent *t*-test (for normally distributed data) or the Mann-Whitney *U*-Test (for non-normally distributed data). The Lilliefors test was used to verify the normality of the distribution of differences between sample populations. To adjust for the risk of error due to multiple comparisons, a Bonferroni correction was applied. Consequently, for the three comparisons made, a *p*-value of less than 0.017 was established as the threshold for defining 95% confidence intervals.

## 3. Results

### 3.1. Study Population

A total of 220 patients were included in this study. The median age was 59.5 [45.2; 65.9] years, and the median BMI was 27.3 [24.0; 30.8] kg/m^2^. The male/female ratio was 131 men (59.5%) to 89 women (40.5%). Most patients were Caucasians, while 24 patients had a different ethnicity. Moreover, 90 patients were previously diagnosed with heart failure, including 39 with a reduced ejection fraction, 17 with a mildly reduced ejection fraction, and 34 with a preserved ejection fraction.

Furthermore, 131 patients were hypertensive, 52 patients had arrhythmias, and 122 patients had valvular diseases, all severities included (95 mitral, 60 aortic). Among the patients with valvular diseases, twenty-one patients had moderate or severe valvular disease: twelve moderate isolated mitral valve regurgitation, with no mitral valve stenosis; eight moderate isolated aortic valve regurgitation; and one severe isolated aortic valve stenosis, with no mixed valve disease and no multiple valve disease. Additionally, among all the patients, 21 had renal failure, and 10 had chronic obstructive pulmonary disease. Examples of complex and heartbeat detection among patients with different cardiovascular diseases are presented in Appendix B.

Kinetic energy parameters and heart rate (HR) (median [Q1; Q3]) assessed by each device and during each cardiac phase (as described in Figure 1) are presented in Table 1. 

### 3.2. Assessments of Repeatability

For each kinetic energy parameter and HR, kinocardiogprah/smartphone and clinician/patient reliability were generated as BA and ICC (Table 2).

**Figure 4:**
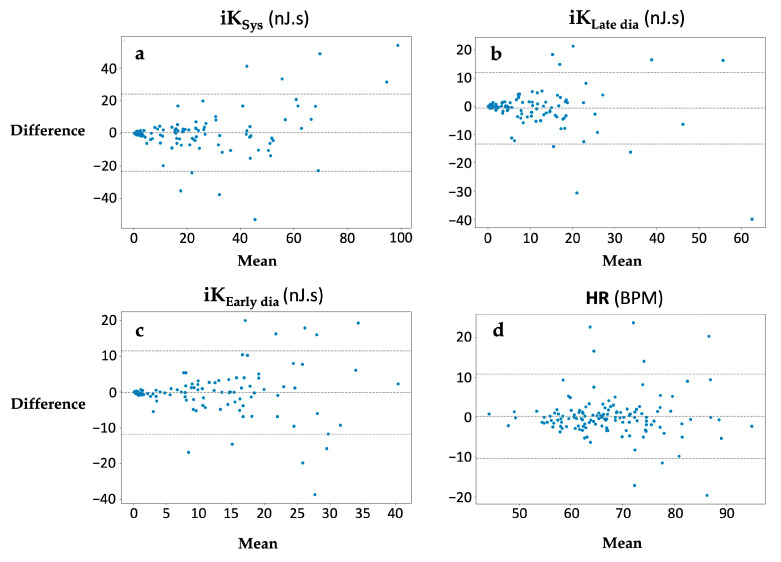
Bland–Altman plot of the difference between (**a**) **iK_Sys_**, (**b**) **iK_Late dia_**, (**c**) **iK_Early dia_**, and (**d**) heart rate (HR) for each subject for the simultaneous kinocardiograph measurement and the smartphone measurement.

The difference between the simultaneous kinocardiograph and smartphone measurements was plotted against the mean of the two measurements. No trends were observed for the computed metrics (Figure 4). The limits of agreement of the compared data were 93.1% for **iK_Sys_**, 93.1% for **iK_Late dia_**, and 92.7% for **iK_Early dia_**. The mean difference in percentage between the two methods for each metric was −15%, −34%, and −1%, respectively (Figure 4). No significant bias was found. The ICC values were 0.86, 0.77, and 0.88. More details and the HR results can be found in Table 2.

**Figure 5:**
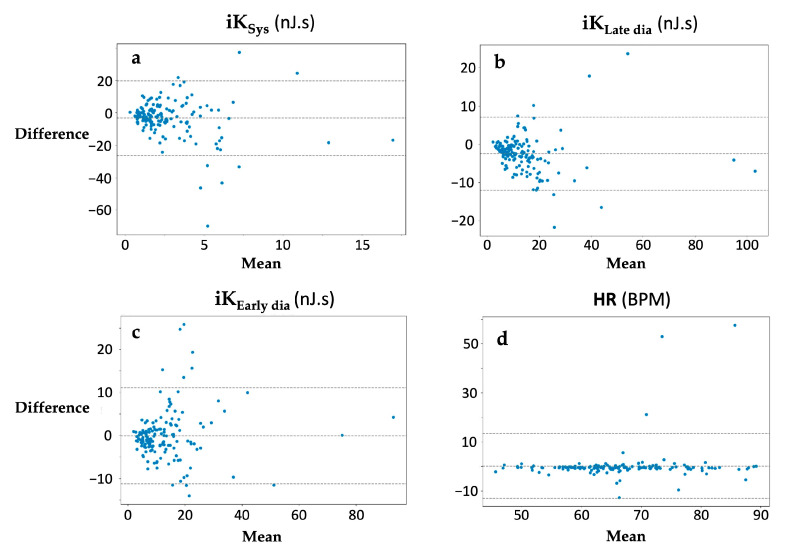
Bland–Altman plot of the difference between (**a**) **iK_Sys_**, (**b**) **iK_Late dia_**, (**c**) **iK_Early dia_**, and (**d**) heart rate (HR) for each subject for the clinician and patient measurements performed both with the patient’s smartphone.

Among 220 patients recruited, 138 had a compatible smartphone. The BA plots for the clinician-patient comparison are shown in Figure 5. No trends were observed for the computed metrics (Figure 5). The limits of agreement of the compared data were 94.9% for **iK_Sys_**, 92.3% for **iK_Late dia_**, and 93.6% for **iK_Early dia_**. The mean difference in percentage between the two measurements for each metric was +3%, −15%, and −3%, respectively (Figure 5). No significant bias was found. The ICC values were 0.85, 0.83, and 0.83. More details, including the HR results and all the 95% confidence intervals (CI), can be found in Table 2. The reliabilities in men and women were in the same ranges. An additional analysis entailed generating the ICC for each metric across various patient subgroups: those with heart failure, hypertension, valvular heart disease, and those without these conditions. The outcomes of this analysis are detailed in Appendix C, Table A2, demonstrating consistent ICC values across diverse cardiac pathologies.

### 3.3. Assessments of Patient Compliance

**Figure 6:**
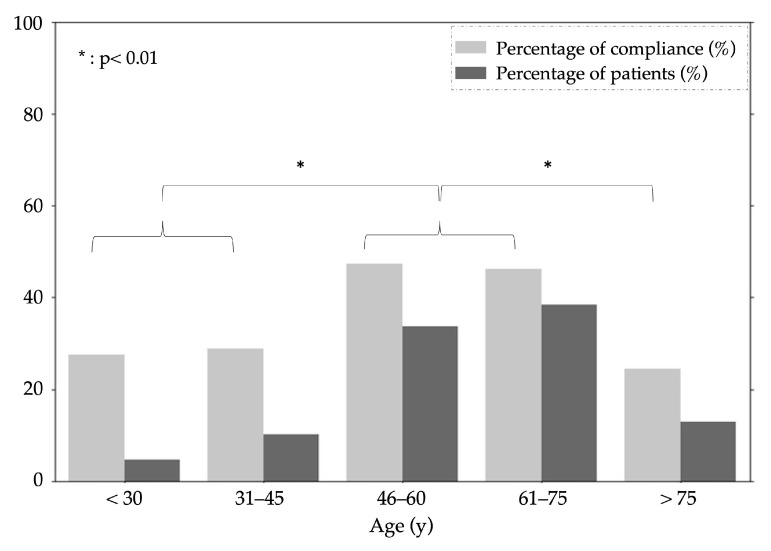
Age-related patient compliance and participation for 138 patients. The graph illustrates the percentage of protocol compliance (dark gray) and the proportion of patient numbers (light gray) across different age categories. Statistically significant differences in compliance are indicated by asterisks (* *p* < 0.01), demonstrating lower adherence in the <46 and >75 age groups compared to the 46–75 age groups.

Out of 220 recruited patients, 138 possessed smartphones that met the specific requirements for the study. These 138 participants were expected to complete a cumulative total of 4968 recordings at home. However, they collectively completed 2058 recordings, resulting in a mean compliance rate of 41.4%. A notable observation from the data was the disparity in recording frequency between different age groups (Figure 6). Specifically, it was noted that patients who were aged between 46 and 75 performed more at-home measurements compared to their younger (+19%) or older (+22%) counterparts (both *p* < 0.01).

## 4. Discussion

### 4.1. Main Findings

The present work evaluated the reliability and reproducibility of automatically computed kinetic energy metrics by comparing a validated device and a commercial smartphone on a representative sample of patients with various cardiovascular diseases. High reliability was found for kinetic energy metrics obtained from smartphone-derived SCG compared to the kinocardiograph. A patient’s capacity to perform a measure on themselves with a commercial smartphone has also been assessed. Our analyses revealed that patients can accurately reproduce the measurement when a clinician teaches them how to make a self-SCG recording with a smartphone. However, this work also confirmed that ease of use was insufficient for a patient to make regular assessments. A continuous and rigorous follow-up is needed to obtain compliance.

### 4.2. Device vs. Commercial Smartphone Reliability and Reproducibility

The approach described aims to simplify cardiac assessment by measuring kinetic energy through body surface accelerations, providing an indirect yet simpler potential alternative to MRI-derived kinetic energy. This method draws inspiration from studies using 4D flow MRI, which have shown promising results in assessing left and right ventricular functions by measuring intra-ventricular kinetic energy. These studies have helped understand the impact of cardiac diseases on intra-cardiac hemodynamics and explore new ways to characterize HF [27,28,29]. Building on these insights, two metrics were developed: the iK systolic (inspired by the LV systolic MRI kinetic energy) and the ΔiK diastolic, reflecting the diastolic phase’s kinetic energy distribution [22]. Differences in iK metrics have been associated with differences in stroke volume, LVEF, and cardiac output in healthy subjects during a dobutamine-induced inotropic increase [30,31] and in heart failure patients [23]. In this work, surrogates to these metrics were described based on an SCG-only acquisition—without ECG—and, in particular, the slicing of the heartbeats based on the sole SCG signal. This allowed the computation of iK from smartphone-derived SCG acquisitions where an ECG is unavailable.

Recently, more interest has been given to smartphone-based cardiac signal acquisition.Mehrang et al. used smartphone-recorded SCG signals to classify cardiac conditions in myocardial infarction patients before and after percutaneous coronary intervention. Involving 20 patients, the study applied noise reduction and feature extraction, followed by classification using random forest and support vector machines. The classifiers achieved 74% and 78% accuracy, respectively, indicating the potential for smartphone-derived cardiac analysis [11].

Another study compared heart rate variability parameters from the smartphone-derived SCG and ECG reference signals. The study involved two groups of subjects, with recordings taken under different conditions. The results demonstrated a strong similarity between RR series extracted from SCG and ECG signals, with differences lower than 10 ms [6].

Smartphone-derived mechanocardiography (sMCG), based on SCG signals combining both linear and angular degrees of freedom [17], has shown promising results in various clinical and research settings [32]. The MODE-AF study focused on using sMCG to detect atrial fibrillation (AF). It enrolled 150 AF patients and 150 controls, using a smartphone-based algorithm for rhythm analysis. The method demonstrated high sensitivity (95.3%) and specificity (96.0%) in distinguishing AF from sinus rhythm, highlighting the potential of smartphones in cost-effective AF screening without additional hardware [12]. In another study, they used sMCG for detecting concurrent AF and acute decompensated heart failure (ADHF). The study, involving 300 cardiac patients, applied machine learning, demonstrating high accuracy in AF detection (area under the curve value of 0.98) with sensitivity and specificity over 90% and moderate accuracy for ADHF detection (0.85 area under the curve) [13].

Albrecht et al. conducted a literature search and a Semi-Automatic Retrospective App Store Analysis to assess SCG applications using smartphones in health and fitness. Despite the technical interest, they found limited practical applications of SCG in smartphone apps, with few research-backed apps in the field [14].

Thus, the results of studies on smartphone-derived SCG applications in cardiovascular disease are encouraging. However, to our knowledge, there is no formal, thorough comparison between a validated device and a smartphone-derived SCG. Our study demonstrates that kinetic energy metrics derived from SCG computed on signals acquired with a device validated in previous studies [16,22] are comparable with an ICC on average greater than 0.8, as well as BA without trends and 92% within the limit of agreements for the different metrics. It is noteworthy that the measurement **iK_Late dia_** demonstrated the most significant level of variability, nearly surpassing the predefined boundaries of the limits of agreement. **iK_Late dia_** is hypothesized to reflect the late diastolic filling associated with the lower energy level [28], which could account for the observed increased variability. In a previous study, kinetic energy metrics derived from SCG computed in healthy subjects on signals acquired 10 to 15 min apart in a supine position were repeatable with an ICC on average greater than 0.85 and CV lower than 5.5% [33]. This finding is consistent with the results of the present study. Therefore, we believe that our findings, performed on a large number of patients with cardiovascular diseases, show that kinetic energy can be computed based on smartphone-derived SCG with high reliability. Furthermore, when computed per cardiac pathology, the ICC showed not to be impeded by diverse cardiac pathologies, as detailed in Appendix C, Table A2.

### 4.3. Clinician vs. Patient Smartphone-Derived SCG Recording

Smartphones have become key in telemedicine for at-home health tracking, enabling patients to monitor vital metrics. However, the reliability of the data can be uncertain when measurements are self-performed, raising concerns about accuracy and interpretation. This emphasizes the need for user education and advanced algorithms to validate data and ensure effective remote healthcare delivery. A study on sMCG evaluated the effectiveness of self-applied smartphones in detecting atrial fibrillation [15]. It involved 182 elderly patients, comparing physician-applied and self-applied sMCG. The knowledge-based learning approach achieved a sensitivity of 96.3% and a specificity of 98% for physician-applied measurements and a 97.6% sensitivity with a 92.9% specificity for self-applied measurements. The best machine learning classifier showed similar performance, indicating the potential of sMCG for reliable AF detection in self-applied settings. Our study demonstrates that kinetic energy metrics derived from SCG computed on signals acquired by a trained clinician or a patient are repeatable with an ICC on average greater than 0.83, as well as BA without trends and 94% within the limit of agreements. Such an accessible tool with a highly reliable measure performed by the patients themselves could open the door to throughout-the-day variations of hemodynamic parameters. Indeed, it is known that BP has a diurnal pattern, and it has been suggested that circadian patterns and night-time blood pressure values may be more highly correlated with indices of end-organ damage than resting blood pressure values [34]. While this phenomenon was extensively studied due to the availability of ambulatory blood pressure recordings [35], the sensitivity of kinetic energy to other cardiovascular parameters, more correlated with stroke volume, may open the possibility of studying new diurnal patterns predicting cardiovascular failure. In Configuration B, where we used the patient’s own smartphone, the results differed from those in Configuration A, which involved the kinocardiograph and a designated study smartphone. This variation primarily stems from the lack of initial quality assessment of the patient’s smartphone, particularly its accelerometer’s accuracy for quantitative measurements. While this discrepancy does not limit our current study, it underscores the importance of thorough quality evaluation for any smartphone used in future applications of this technique, especially when using a patient’s personal device.

In the context of cardiovascular patient demographics, a significant portion of this population comprises older individuals. These patients often face economic constraints and prefer traditional mobile phone technology, factors which may impede the adoption of this technique. Within the scope of this research, it was observed that only 62.7% of the participants possessed a smartphone capable of supporting the required application for SCG acquisition. Moreover, adherence to the protocol was noted at 41.4%, showing a decline when patients were younger than 45 or older than 75, despite a dedicated study nurse’s proactive engagement through regular telephonic reminders. The decline in adherence among the youngest and oldest age groups could be attributed to various barriers. For younger patients, these might include lifestyle factors, such as busier schedules or lack of perceived need. In contrast, older patients might face challenges like physical limitations or cognitive decline. Identifying and addressing these barriers is essential for improving adherence. The findings highlight the need for more personalized approaches in patient care and protocol adherence strategies. Tailoring interventions to cater to the specific needs and circumstances of different age groups could potentially enhance compliance rates. Specifically for the elderly demographic, this raises concerns about the feasibility of smartphone-based self-monitoring. Potential strategies include an adaption of the measurement frequency according to the pathology severity and the involvement of a third party, such as a family physician, nurse, or family member, to assist in smartphone operations. Additionally, patient compliance is anticipated to improve following the clinical validation of this technique and its endorsement by the medical community.

### 4.4. Future Directions and Perspectives

Smartphone-based monitoring for heart failure decompensation in chronic patients could offer detailed tracking of symptoms and vital signs at home. This approach would enable early detection of exacerbations, allowing for quicker treatment adjustments. As a result, it has the potential to significantly reduce rehospitalization rates by preventing HF from worsening, among other potential applications. Consistent and accurate data collection through smartphones could enhance patient engagement and allow healthcare providers to make timely, informed decisions, ultimately improving patient outcomes and reducing the strain on healthcare systems.

The reliance on patient-reported data requires accuracy in measurement and interpretation for effective remote healthcare. This research is designed to present initial findings for this specific challenge. Furthermore, a previous paper evaluated kinocardiography for monitoring fluid status in 11 heart failure patients, showing that cardiac kinetic energy increases significantly due to fluid status changes during diuretic treatment. This suggests the potential of SCG-derived kinetic energy as a non-invasive monitoring tool for detecting subtle hemodynamic changes in heart failure patients [36].

However, it is noteworthy that integrating smartphone-based medical data acquisition within the regulatory framework of the Medical Device Regulation (MDR) presents challenges. The MDR demands rigorous validation of medical devices to ensure safety and effectiveness, which includes software used in decision-making. For smartphone applications, this involves proving accuracy, reliability, and data security, adhering to strict standards for clinical evidence and risk management.

### 4.5. Limitations

The present work also presents some limitations worth noting. The kinetic energy computation method introduced in this study differs from those detailed in earlier publications. Consequently, it is essential to validate this new method to ensure its comparability with the previously established methods. Additionally, this approach uses fixed window lengths centered on SCG peak detection to determine the different phases. Developing adaptive window lengths based on variables like heart rate could enhance the accuracy of the identified cardiac phases [37]. Moreover, this study did not assess the reliability of the proposed method’s beat-by-beat heartbeat detection. While the reliability of the kinetic energy metrics suggests the detections were acceptable, a comprehensive analysis of detection performance could enhance the technique’s reliability and enable comparisons with similar methods [38,39]. As presented in the methods, the reliability of rotational kinetic energy was not investigated in the current work; therefore, the results obtained here are only relevant to linear kinetic energy. Previous studies [40,41] have established that respiration markedly influences the metrics obtained from SCG. However, this study did not investigate the repeatability of metrics derived from a specific respiration phase. This aspect presents a potential interest for future research. The kinocardiograph device and the commercial smartphone were placed next to each other to obtain simultaneous signals. Therefore, the sensors were not acquiring signals from the same position, which may have induced a difference in the accelerations acquired between the two devices [42,43]; however, they are of small amplitude, given the BA results. When returned home for phase C, patients were not given specific anatomical landmarks for smartphone placement. Instead, they were simply advised to position the smartphone on their sternum, mirroring the clinician’s method as closely as possible. Providing more precise instructions in the future could potentially enhance the accuracy of the results. For phases B and C, the patients’ smartphones were used, including different brands and models. The placement of the inertial unit within the enclosure varies among these different smartphones. This was not taken into account in this study to compensate for the sampling point, providing an additional point that could improve the reliability of these measurements. This research confirmed that kinetic energy metrics, specifically derived from SCG and acquired via a smartphone, are both obtainable and repeatable when performed by the patients themselves. Nevertheless, it is worth pointing out that this validation is specific to the kinetics energy metrics in the context of SCG rather than SCG as a whole.

## 5. Conclusions

This research confirms the feasibility of acquiring kinetic energy metrics through smartphone-based SCG and establishes their repeatability when conducted by patients. Notably, this validation was achieved through the active participation of patients specifically recruited from the cardiology ambulatory department, thus reinforcing the relevance of the results in the field of cardiovascular telemedicine. The observed high accuracy distinctly highlights the potential utility of smartphone-derived seismocardiography as a reliable and accessible tool for cardiovascular assessment. While patient compliance remains a significant challenge, this research lays the groundwork for subsequent validations. These validations could potentially illustrate the benefits of at-home, smartphone-based SCG acquisition in improving the quality of life for individuals suffering from cardiac conditions.

## Figures and Tables

**Table 1:** Kinetic energy parameters and average heart rate (HR) (median [Q1; Q3]) for configuration A—Device and Smartphone—and configuration B: Healthcare professional (HCP) recording with the patient’s smartphone and patient recording with the patient’s smartphone.

Measurements	iK_Sys_ (nJ·s)	iK_Late dia_ (nJ·s)	iK_Early dia_ (nJ·s)	HR (bpm)
Kinocardiograph (A)	18.6 [5.9; 55.7]	7.4 [2.6; 26.9]	10.1 [3.1; 32.2]	64.7 [51.3; 85.0]
Reference Smartphone (A)	21.6 [7.9; 70.0]	10.0 [2.9; 39.4]	10.0 [3.8; 27.7]	65.6 [51.5; 82.9]
Patient’s Smartphone by HCP (B)	11.2 [0.6; 70.7]	4.3 [0.2; 27.4]	6.8 [0.2; 29.7]	66.1 [54.2; 84.0]
Patient’s Smartphone by Patient (B)	13.5 [0.6; 59.9]	4.9 [0.2; 30.5]	6.2 [0.2; 31.3]	65.9 [54.7; 83.4]

**Table 2:** Commercial smartphone vs. device comparison and clinician vs. patient measures are compared as Bland–Altman Bias (BA Bias (mean [CI 95%]) (nJ**·s**)), Bland–Altman limits of agreement (BA LoA (nJ**·s**)) and intraclass correlations coefficients (${ICC}_{c}$ (mean [CI 95%])) for kinetic energy parameters and heart rate (HR).

	Features	iK_Sys_	iK_Late dia_	iK_Early dia_	HR
Device Comparison (A)	BA Bias ([95% CI]) (nJ**·s**)	0.01 [−0.05; 0.015]	−0.08 [−0.13; 0.01]	−0.01 [−0.04; 0.013]	0.03 [−0.04; 0.09]
	BA LoA [Lower; Upper] (nJ**·s**)	[−22; 22]	[−12; 11]	[−11; 11]	[−10; 11]
	${ICC}_{c}$ ([95% CI])	0.86 [0.81; 0.90]	0.77 [0.61; 0.85]	0.88 [0.84; 0.91]	0.77 [0.7; 0.83]
Patient vs. clinician (B)	BA Bias ([95% CI]) (nJ**·s**)	−2 [−2.50; −0.50]	−3 [−4.20; −2.10]	−0.01 [−0.03; 0.011]	0.03 [−0.03; 0.10]
	BA LoA [Lower; Upper] (nJ**·s**)	[−25; 20]	[−12; 7]	[−11; 10]	[−13; 13]
	${ICC}_{c}$ ([95% CI])	0.85 [0.80; 0.89]	0.83 [0.77; 0.87]	0.83 [0.77; 0.87]	0.83 [0.77; 0.88]

**Figure A1:**
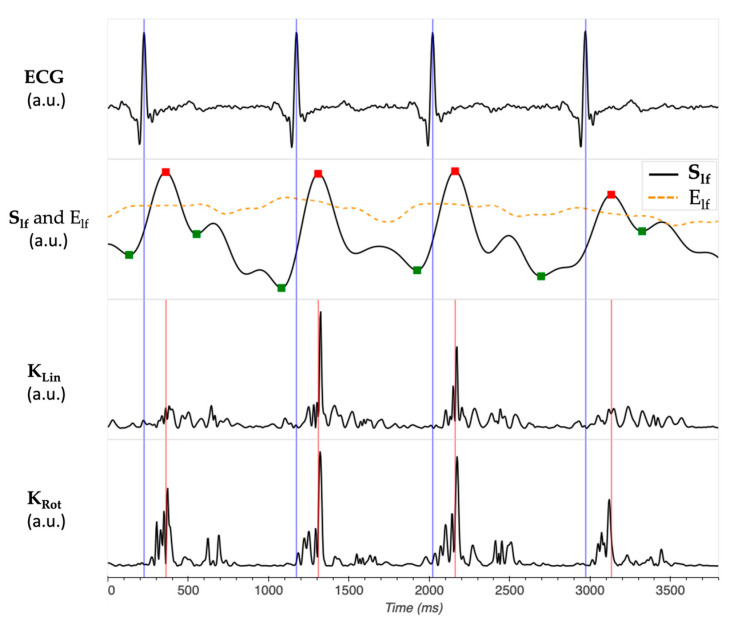
Patients recruited during the study with no signs of heart failure, hypertension, or valvular heart disease. The plots shown represent, from top to bottom, the ECG from the kinocardiograph, a combination of the linear and rotation low-frequency signals, namely the Slf, the RMS envelope of Slf, namely Elf, and finally, the kinetic energy signals in the linear and rotational dimensions, respectively KLin and KRot.

**Figure A2:**
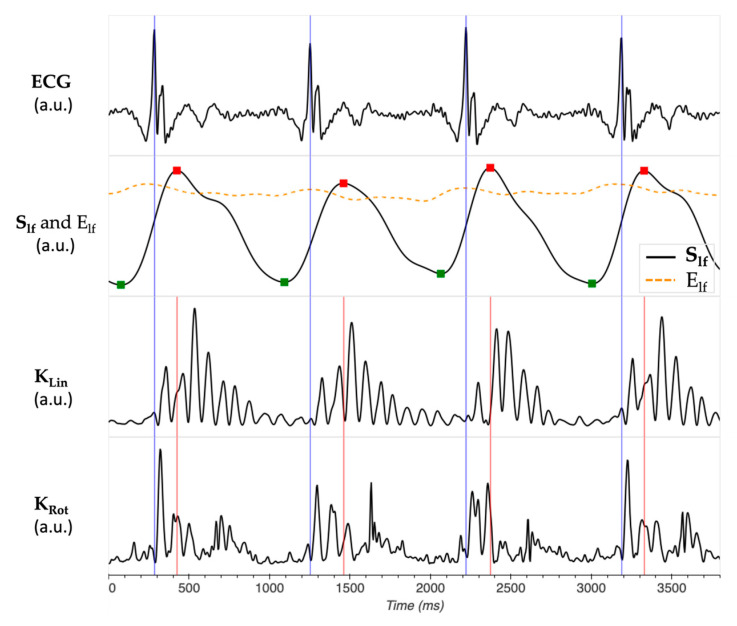
Patients recruited during the study with hypertension and moderate aortic insufficiency. The plots shown represent, from top to bottom, the ECG from the kinocardiograph, a combination of the linear and rotation low-frequency signals, namely the Slf, the RMS envelope of Slf, namely Elf, and finally, the kinetic energy signals in the linear and rotational dimensions, respectively KLin and KRot.

**Figure A3:**
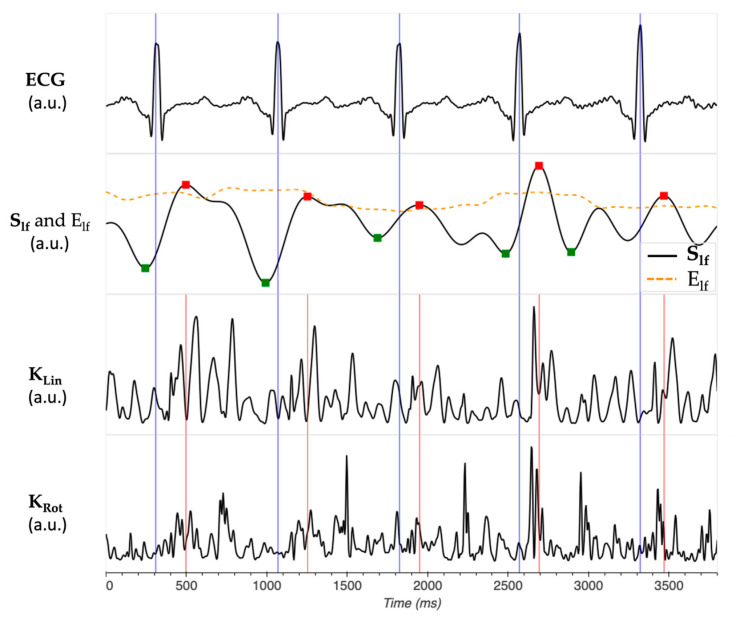
Patients recruited during the study with heart failure. The plots shown represent, from top to bottom, the ECG from the kinocardiograph, a combination of the linear and rotation low-frequency signals, namely the Slf, the RMS envelope of Slf, namely Elf, and finally, the kinetic energy signals in the linear and rotational dimensions, respectively KLin and KRot.

## Data Availability

The datasets used and analyzed during the current study are available from the corresponding author upon reasonable request.

## Appendix A

In the scope of this work, the clinical intervals are called agreement intervals. The agreement intervals were determined based on the variation observed between patients with heart failure vs. a matched control group, as reported in a previous study [22]. Consequently, the agreement intervals for each metric are a 67% variation in iK_Sys_, 35% in iK_Late dia_, and 7% in iK_Early dia_ in terms of percentage change; see Table A1. Therefore, measurements were considered comparable when the limits of agreement, defined as ±1.96 standard deviations between both measurements with a 95% Confidence Interval, aligned within these pre-established clinical agreement intervals.

**Table A1:** Variations observed in kinetic energy metrics derived from seismocardiography between patients with heart failure vs. a matched control group, as reported in a previous study [22].

Measurements	Heart Failure	Control
**iK_Sys_ (μJ·s)**	190 [85; 800]	570 [80; 1000]
**iK_Late dia_ (%)**	0.205 [0.11; 0.35]	0.135 [0.09; 0.15]
**iK_Early dia_ (%)**	0.805 [0.65; 0.89]	0.865 [0.85; 0.91]

## Appendix B

Figure A1, Figure A2 and Figure A3 were drawn to illustrate the method described in Section 2.2. to detect peaks and compute kinetic energy on the SCG signal. For each patient, the plots shown represent the ECG from the kinocardiograph, a combination of the linear and rotation low-frequency signals, namely the S_lf_, the RMS envelope of S_lf_, namely E_lf_, and finally, the kinetic energy signals in the linear and rotational dimensions, respectively K_Lin_ and K_Rot_. Figure A1 represents a patient without heart failure, hypertension, or valvular heart disease. The peak detection and different phases of the kinetic energy signals are particularly visible and visually identifiable. Conversely, Figure A2 illustrates a hypertensive patient’s data, where, despite identifiable heartbeats, peak identification proves difficult; however, the amalgamation of linear and rotational signals facilitates potential complex center position determination. Figure A3 features a heart failure patient, presenting challenges in identifying the principal complex and its central peak. Nonetheless, the applied method appears to maintain the complex’s identification in alignment with the ECG peak despite the beat-to-beat variability of the potential central peak.

This illustrates that, although Appendix C demonstrates consistent intraclass correlation coefficient (ICC) values across varying pathologies—underscoring the method’s repeatability across different devices and execution by either clinician or patient—the fundamental performance in complex and peak detection, particularly within subjects exhibiting advanced cardiac conditions, warrants further investigation.

## Appendix C

Additional analysis entailed generating the ICC for each kinetic energy parameter and heart rate, assessing kinocardiograph-smartphone and clinician-patient reliability across various patient subgroups: those with heart failure (90 patients), hypertension (131 patients), valvular heart disease (all severities included, 122 patients), and those without these conditions (51 patients). The outcomes of this analysis are detailed in Table A2, demonstrating consistent ICC values across diverse cardiac pathologies.

**Table A2:** Commercial smartphone vs. kinocardiograph comparison and clinician vs. patient measures are compared as intraclass correlation coefficients (${ICC}_{c}$ (mean [CI 95%])) for kinetic energy parameters and heart rate (HR). HF: heart failure; HT: hypertension; VHD: valvular heart disease; OTHER: patient without these conditions.

	Measurements	iK_Sys_	iK_Late dia_	iK_Early dia_	HR
Device Comparison (A)	${ICC}_{c}$ HF ([95% CI])	0.87 [0.77; 0.93]	0.84 [0.60; 0.93]	0.96 [0.94; 0.98]	0.83 [0.77; 0.94]
	${ICC}_{c}$ HT ([95% CI])	0.90 [0.85; 0.93]	0.78 [0.54; 0.89]	0.80 [0.72; 0.86]	0.81 [0.74; 0.87]
	${ICC}_{c}$ VHD ([95% CI])	0.86 [0.79; 0.91]	0.82 [0.57; 0.91]	0.89 [0.85; 0.92]	0.70 [0.60; 0.78]
	${ICC}_{c}$ OTHER ([95% CI])	0.89 [0.83; 0.93]	0.80 [0.52; 0.90]	0.84 [0.77; 0.89]	0.97 [0.95; 0.98]
Patient vs. clinician (B)	${ICC}_{c}$ HF ([95% CI])	0.81 [0.65; 0.90]	0.78 [0.60; 0.89]	0.80 [0.62; 0.90]	0.75 [0.55; 0.87]
	${ICC}_{c}$ HT ([95% CI])	0.86 [0.80; 0.91]	0.84 [0.77; 0.90]	0.81 [0.73; 0.88]	0.83 [0.76; 0.89]
	${ICC}_{c}$ VHD ([95% CI])	0.84 [0.78; 0.89]	0.83 [0.77; 0.88]	0.81 [0.74; 0.87]	0.83 [0.76; 0.88]
	${ICC}_{c}$ OTHER ([95% CI])	0.83 [0.74; 0.89]	0.85 [0.78; 0.91]	0.82 [0.73; 0.89]	0.80 [0.70; 0.87]
